# Next generation sequencing panel as an effective approach to genetic testing in patients with a highly variable phenotype of neuromuscular disorders

**DOI:** 10.1007/s10048-024-00762-y

**Published:** 2024-05-17

**Authors:** Wiktoria Radziwonik-Fraczyk, Ewelina Elert-Dobkowska, Marek Karpinski, Jacek Pilch, Karolina Ziora-Jakutowicz, Jolanta Kubalska, Dominika Szczesniak, Iwona Stepniak, Jacek Zaremba, Anna Sulek

**Affiliations:** 1 Department of Genetics, Institute of Psychiatry and Neurology, Warsaw, Poland; 2 Specialist Hospital John Paul II, Cracov, Poland; 3 Department of Pediatric Neurology, Medical University of Silesia, Katowice, Poland; 4 Faculty of Medicine, Lazarski University, Warsaw, Poland

**Keywords:** Neuromuscular disorders, Next-generation sequencing, Targeted gene panel, MLPA analysis

## Abstract

Neuromuscular disorders (NMDs) include a wide range of diseases affecting the peripheral nervous system. The genetic diagnoses are increasingly obtained with using the next generation sequencing (NGS). We applied the custom-design targeted NGS panel including 89 genes, together with genotyping and multiplex ligation-dependent probe amplification (MLPA) to identify a genetic spectrum of NMDs in 52 Polish patients. As a result, the genetic diagnosis was determined by NGS panel in 29 patients so its diagnostic utility is estimated at 55.8%. The most pathogenic variants were found in *CLCN1*, followed by *CAPN3*, *SCN4A*, and *SGCA* genes. Genotyping of myotonic dystrophy type 1 and 2 (DM1 and DM2) as a secondary approach has been performed. The co-occurrence of *CAPN3* and *CNBP* mutations in one patient as well as *DYSF* and *CNBP* mutations in another suggests possibly more complex inheritance as well as expression of a phenotype. In 7 individuals with single nucleotide variant found in NGS testing, the MLPA of the *CAPN3* gene was performed detecting the deletion encompassing exons 2—8 in the *CAPN3* gene in one patient, confirming recessive limb-girdle muscular dystrophy type 1 (LGMDR1). Thirty patients obtained a genetic diagnosis (57.7%) after using NGS testing, genotyping and MLPA analysis. The study allowed for the identification of 27 known and 4 novel pathogenic/likely pathogenic variants and variants of uncertain significance (VUS) associated with NMDs.

In conclusion, the diagnostic approach with diverse molecular techniques enables to broaden the mutational spectrum and maximizes the diagnostic yield. Furthermore, the co-occurrence of DM2 and LGMD has been detected in 2 individuals.

**Supplementary Information:**

The online version contains supplementary material available at 10.1007/s10048-024-00762-y.

## Introduction

Neuromuscular disorders (NMDs) are a highly heterogeneous group of inherited disorders characterized by the impairment of skeletal and heart muscles, peripheral nerves, neuromuscular junctions, and spinal cord motor neurons, leading to muscle weakness and/or atrophy, hypertrophy, pseudohypertrophy and fatty infiltration [1, 2]. Their classification is based on the site of the pathology [3]. More than 600 genes related to NMDs have been identified [2], and their number is still growing. In addition, their phenotypic complexity depends on various circumstances: the occurrence of myopathic and neurogenic findings in different members of one family [4], different phenotypes in a single patient [5, 6], or even two separate conditions in a single patient can be present [7]. It makes NMDs diagnosis more challenging and, therefore, high-throughput next generation sequencing (NGS) technology, enabling massive parallel sequencing of many genes simultaneously, is increasingly used in clinical settings. The most common approaches in NMD genetic diagnostics comprise targeted gene panel (TGP), including clinical exome, and/or whole exome sequencing (WES). It has been revealed that the diagnostic yield of TGP may differ from 15.1% to even 49.3%, depending on the number of study groups and the number of analyzed genes [8–11]. However, there is a possibility to increase the utility by using a comprehensive TGP, which involves all updated known disease-causing genes with high coverage of these target genes [12]. Barbosa-Gouveia et al. (2022) confirmed that increasing the number of causative genes from 278 to 324 enables to obtain a higher diagnostic rate from 31 to 40% in 268 NMD patients [13]. On the contrary, the increasing number of investigated genes has not always led to a significant rise in diagnosed patients [14]. At the same time, WES facilitates identifying novel disease-causing genes. Its utility ranges from 26% [15] to 39% [16]. It is noteworthy that diagnostic reassessment and variant reclassification after using WES enabled maximizing a diagnostic rate [12]. Regardless of the NGS approaches, still many patients remain undiagnosed genetically, which leads to diagnostic delays. In diagnostic practice, a well-designed gene panel, deep NGS coverage [17], a combination of different molecular techniques, and reanalysis of NGS data with a detailed clinical assessment of patients regarding updated knowledge in the literature and databases [18] may lead to increased diagnostic effectiveness and meet objectives of genetic diagnostics.

To identify the genetic cause of NMDs, we combined different molecular biology techniques, such as NGS, fragment analysis, genotyping and multiplex ligation-dependent probe amplification (MLPA) assays. We aimed to: (1) assess the diagnostic utility of a custom-designed 89 gene panel; and (2) describe unexpected findings in selected patients with clinical features of NMDs.

## Materials and methods

### Study design

The presented NGS panel for 89 genes has been developed to diagnose patients with neuromuscular diseases referred to the Genetic Clinic, the Institute of Psychiatry and Neurology (IPiN). The genes involved in pathological mechanisms of muscular dystrophies, myopathies and myotonic syndromes have been selected according to several sources. The PubMed browser was searched according to the [neuromuscular disorders] and [genetic testing] terms to look for the most relevant genes in diagnostics. In addition, Neuromuscular Disease Center website [19], GeneReviews, and OMIM database were screened to specify the list of the genes (Supplementary Table 1). Due to the inability of panel’s to identify microsatellite repeats, highly homologous regions, regions of high/low GC content and variable sensitivity to detect copy number variations (CNVs), MLPA as well as DM1 and DM2 expansion analysis were carried out [20]. This panel has been used in our laboratory for 2 years. Although, for future diagnostic patients with clinical diagnosis of NMD, a panel expanded to 244 genes embracing genes implicated in mitochondrial disorders, neuropathies and very rare types of muscular dystrophies, myopathies, or myasthenia will be performed. The effectiveness and relevance of the updated panel will be assessed after examining a numerically similar group of patients.

### Patients

A total number of 52 unrelated Polish patients including 23 females (44.2%) and 29 males (55.8%) with clinically suspected NMDs were enrolled in the study. The disease onset ranged from infancy to late adulthood (3 months – 63 years). Patients were successively referred by clinicians from genetic counselling from all over Poland for further genetic testing towards NMDs because these patients were not diagnosed genetically before this study. The neurological description was based on an available medical history (assessing weakness, location and anatomic distribution of neuromuscular symptoms and accompanied features, onset, and course of the disease, family history), clinical assessment of muscle weakness, fatigability, myopathy. Numerous studies including laboratory tests such as assessment creatine kinase (CK), aspartate aminotransferase (AST), alanine aminotransferase (ALT) levels, general urine and cerebrospinal fluid examination, visual evoked potentials testing, magnetic resonance imaging of the brain and brainstem, needle electromyography (EMG)/motor nerve conduction examination, motor unit analysis or muscle biopsy (if available) were performed in selected patients. The limitation of our study was incomplete data of CK levels. Data on CK levels and/or muscle biopsy were not available for all patients. Typically, patients diagnosed with dystrophy were first analyzed for deletion/duplication in the *DMD* gene causing Duchenne/Becker muscular dystrophy, and less frequently for Pompe disease, while patients diagnosed with myotonia underwent genotyping for DM1 and/or DM2. Exclusion of these diseases made it possible to conduct a study using 89 NMD gene panel. However, for patients referred from other centers than Institute of Psychiatry and Neurology (IPiN), DM1 and DM2 genotyping was performed after using gene panel.

The control group involved 172 DNA samples derived from Polish patients who were referred to our department with non-related NMD phenotype and in whom NGS testing was performed. All genetic variants detected in this group were reported in internal IPiN database, which is used to assess the frequency of particular variants found in NMD patients regarding control groups as well as for reevaluation of variants of uncertain significance (VUS). The study was approved by the Ethics Committee of the IPiN in Warsaw, Poland. All participants gave the written informed consent, including patients under 18 years (for whom the consent was signed by parents) as well as family members of affected patients, in whom segregation analysis was done.

### Methods

Genomic DNA was extracted from peripheral blood using the MagNA Pure Compact Nucleic Acid Isolation Kit I – Large Volume (Roche), following the manufacturer’s instructions. The quantity and quality of the isolated DNA were assessed by UV/VIS Spectrophotometer NanoDrop 2000 (Thermo Fisher Scientific) and Qubit fluorometer (Invitrogen, Thermo Fisher Scientific).

#### Targeted next generation sequencing and data analysis

In the present study, TGP covered exons and intron/exon boundaries (+ 50 bp) of 89 genes involved in muscular disorders, including muscular dystrophy (34 genes), congenital muscular dystrophy (22 genes), congenital myopathy (21 genes), distal myopathy (16 genes), myofibrillar myopathy (9 genes), myotonic syndromes (6 genes), periodic paralysis (3 genes), congenital myasthenic syndromes (13 genes), Emery-Dreifuss muscular dystrophy (6 genes), limb-girdle muscular dystrophy (24 genes), was designed.

The patient DNA library was prepared from 250 ng genomic DNA with a KAPA HyperPlus Kit (Roche) and sequencing of the NGS libraries was performed by a MiSeq (Illumina) paired-end 2 × 75-bp DNA sequencing platform with a MiSeq Reagent Kit v3 (150 cycles), according to the manufacturer’s instructions. Quantification analysis and assessment of the average size and length of the NGS libraries were performed using a Bioanalyzer assay (Agilent).

The analysis of the enriched sequencing data was performed for a minimum target of coverage at 50X. Furthermore, minimum coverage of 20X was required for at least 95% of the targeted sequence.

The analysis of gene variants was performed using BaseSpace Variant Interpreter, and the interpretation was made according to the American College of Medical Genetics and Genomics (ACMG) and the Association for Molecular Pathology (AMP) Standards and Guidelines [21]. ACMG nomenclature guidelines were applied for naming of all genetic variants. The analysis was conducted based on the human reference genome hg19. The initial variant filtering included the following criteria: (1) all coding consequences (stop gain or loss, splice site, indels, missense, and protein altering), (2) gnomAD frequency value less than 2% for all populations, and (3) small variant quality control (QC) metrics with value > 35% for variant read frequency. To investigate the functional predictions of the variants, several in silico algorithms were used: CADD (https://cadd.gs.washington.edu/snv) as well as Revel, DANN, MetaLR, SIFT, and PolyPhen2 for evaluation of single-nucleotide substitutions or SpliceAI for splice-site variants. MutationTaster (http://www.mutationtaster.org/) was also carried out. To assess the clinical significance of DNA variants, we used ClinVar, LOVD (Leiden Open Variation Database), Franklin database (https://franklin.genoox.com/clinical-db/home) and Mastermind Genomic Search (https://www.genomenon.com/mastermind/), whereas the population frequency of the variants was determined by gnomAD v2.1.1 and v3.1.2 (Genome Aggregation Database). The prevalence of selected variants was also compared between NMD patients and the control individuals using internal database. The additional assessment of the quality of NGS data was performed using a genome visualization tool—Integrative Genomics Viewer (IGV).

#### Sanger sequencing

All pathogenic/likely pathogenic variants identified by NGS and consistent with the clinical phenotype of the patients were confirmed by Sanger sequencing on an ABI 3130 genetic analyzer (*Applied Biosystems*). The segregation analysis to assess the pathogenicity of variants classified as pathogenic/likely pathogenic and VUS was performed in the case when DNA samples of family members (both affected and unaffected) were available.

#### Multiplex ligation-dependent probe amplification analysis/assays for microrearrangements detection

Four homozygous and three heterozygous carriers of the *CAPN3* gene were screened for the detection of all exon deletions and/or duplications in this gene using SALSA MLPA Probemix P176-C3 kit (MRC Holland, Netherlands) and following manufacturer’s protocols. MLPA data were analyzed with Coffalyser.Net™ Software (MRC Holland, Netherlands).

#### Genotyping for dynamic mutation detection

The genotyping was performed to test the presence of heterozygous microsatellite repeat expansion (CTG)_n_ in the *DMPK* gene (myotonic dystrophy type 1, DM1) and a heterozygous expansion of the CCTG repeat in the *CNBP* gene (myotonic dystrophy type 2, DM2). The PCR reaction was performed as described elsewhere [22, 23]. Additional tests based on the repeat primed PCR (RP_PCR) were performed in cases with the only one allele observed in basic reaction. Analysis of the PCR products were performed after capillary electrophoresis on ABI 3130 genetic analyzer (*Applied Biosystems*) to detect the presence of expanded alleles. DM1 was analyzed in 51, whereas DM2 in 52 individuals.

## Results

**Fig. 1:**
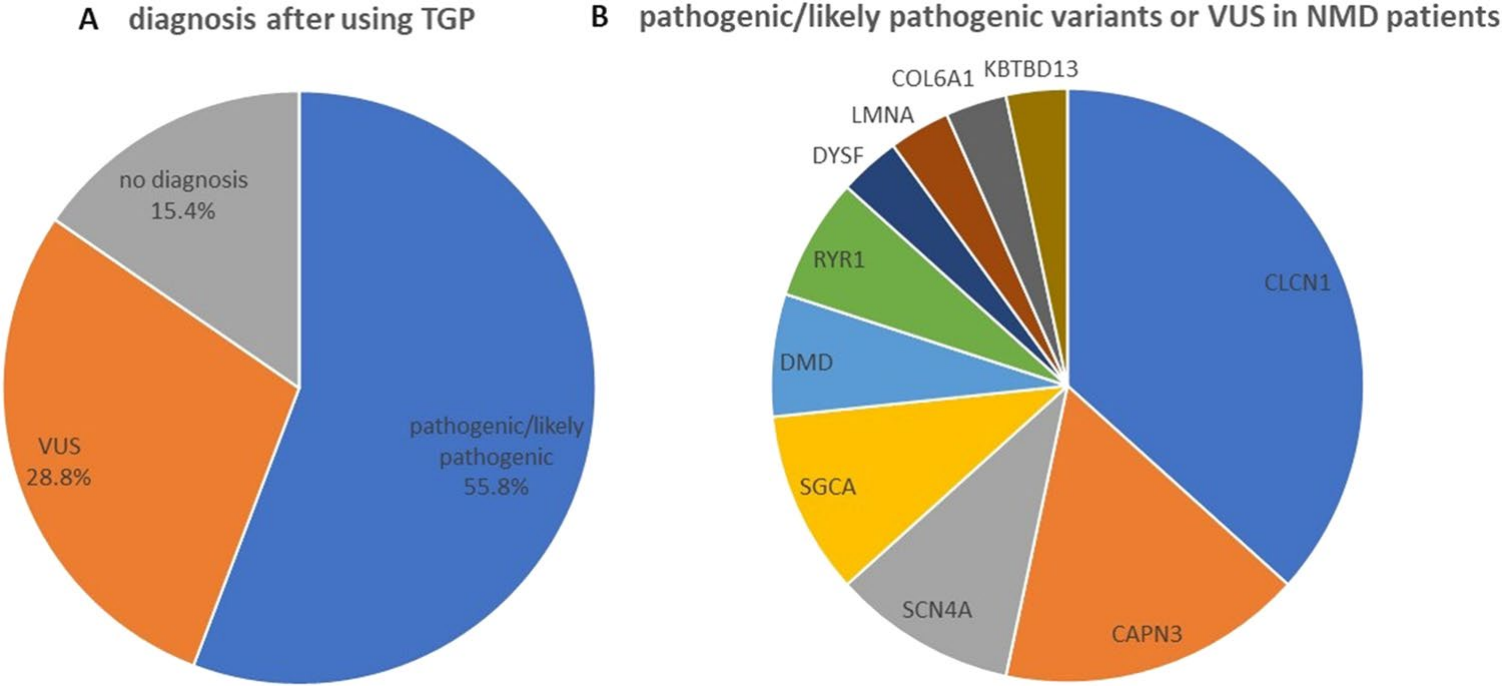
**A** The frequencies of patients with detected pathogenic/likely pathogenic variants, VUS, and still undiagnosed patients after using TGP in our Polish group of 52 patients. **B** The frequency of pathogenic, likely pathogenic variants, and VUS in particular genes causing NMDs

In total, 52 patients with an initial diagnosis of NMDs were included in this study. Thirty of them obtained a genetic diagnosis (57.7%) after being tested with the use of 89 gene panel, genotyping and MLPA analysis (Fig. 1A).

### Coverage and depth of sequencing

Enrichment sequencing data showed an average coverage depth of 116.2X (maximum value was 170X and minimum – 79.5X), with the average uniformity of coverage (Pct > 0.2*mean) of 97.1% (maximum value was 97.7% and minimum – 95.9%). The average depth of sequencing coverage at 20X was 97.5%, ranging from 95.1% to 98.4%. The 20X sequence coverage was obtained for an average 97.5% of targeted sequence (from 95.1% to 98.4%).

### NGS results

As a result of using TGP associated with 89 NMD − causing genes, 56 different variants including pathogenic, likely pathogenic, and VUS have been identified (Table 1).

**Table 1:** Overview of molecularly confirmed 30 patients with clinical diagnosis of neuromuscular disease

Patient ID	Gender	Onset	Age at diagnosis (yrs)	Gene symbol	Reference sequence	Mutation nucleotide (protein)	Genotype	ACMG classification	CADD score*	REVEL score	Novel	Serum CPK levels	Muscle biopsy
Myotonia congenita, autosomal recessive													
P1	M	childhood	21	*CLCN1*	NM_000083.2	c.2680C > T (p.Arg894Ter)	Homozygote	Likely pathogenic (PVS1, PM2, PP5)	35	N/A	No	Not done	Not done
P2	M	16	20	*CLCN1*	NM_000083.2	c.2680C > T (p.Arg894Ter)	Homozygote	Likely pathogenic (PVS1, PM2, PP5)	35	N/A	No	213 U/L	Not done
P3	F	childhood	35	*CLCN1*	NM_000083.2	c.2680C > T (p.Arg894Ter)	Homozygote	Likely pathogenic (PVS1, PM2, PP5)	35	N/A	No	63 U/L	Not done
P4	F	10	36	*CLCN1*	NM_000083.2	c.2680C > T (p.Arg894Ter)	Homozygote	Likely pathogenic (PVS1, PM2, PP5)	35	N/A	No	Not done	Not done
P5	M	childhood	41	*CLCN1*	NM_000083.2	c.2680C > T (p.Arg894Ter)	Homozygote	Likely pathogenic (PVS1, PM2, PP5)	35	N/A	No	Not done	Not done
P6	F	24	29	*CLCN1*	NM_000083.2	c.871G > A (p.Glu291Lys)	Heterozygote compound	Pathogenic (PM3, PP3, PM2, PM1, PP2, PS3, PP1, PP5)	33	Deleterious (Strong)	No	Not available	Not available
						c.2680C > T (p.Arg894Ter)		Likely pathogenic (PVS1, PM2, PP5)	35	N/A	No		
P7	F	Not available	25	*CLCN1*	NM_000083.2	c.899G > A (p.Arg300Gln)	Heterozygote compound	Benign (PP3, PP2, BP6, BS1, BS2)	28.9	Deleterious (Moderate)	No	Not available	Not available
						c.1238 T > G (p.Phe413Cys)		Pathogenic (PS4, PM2, PM1, PP3, PP2, PS3, PP1, PP5)	27.7	Deleterious (Moderate)	No		
P8	F	early childhood	45	*CLCN1*	NM_000083.2	c.899G > A (p.Arg300Gln)	Heterozygote compound	Benign (PP3, PP2, BP6, BS1, BS2)	28.9	Deleterious (Moderate)	No	148 U/L	Not done
						c.1231G > T (p.Gly411Cys)		Pathogenic (PS4, PP3, PM2, PM1, PP2, PP5)	32	Deleterious (Strong)	No		
						c.2680C > T (p.Arg894Ter)		Likely pathogenic (PVS1, PM2, PP5)	35	N/A	No		
P9	M	childhood	18	*CLCN1*	NM_000083.2	c.1238 T > G (p.Phe413Cys)	Heterozygote compound	Pathogenic (PS4, PM2, PM1, PP3, PP2, PS3, PP1, PP5)	27.7	Deleterious (Moderate)	No	Not available	Not available
						c.2680C > T (p.Arg894Ter)		Likely pathogenic (PVS1, PM2, PP5)	35	N/A	No		
P10	F	6	13	*CLCN1*	NM_000083.2	c.1437_1450del (p.Pro480HisfsTer24)	Heterozygote compound	Pathogenic (PVS1, PM3, PM2, PP5)	36	N/A	No	86 U/L	Not done
						c.2680C > T (p.Arg894Ter)		Likely pathogenic (PVS1, PM2, PP5)	35	N/A	No		
P11	M	Not available	30	*CLCN1*	NM_000083.2	c.1697C > T (p.Ala566Val)	Heterozygote compound	Pathogenic (PM3, PP3, PM2, PM1, PP2, PP5)	29.6	Deleterious (Strong)	No	Not available	Not available
						c.2680C > T (p.Arg894Ter)		Likely pathogenic (PVS1, PM2, PP5)	35	N/A	No		
Myotonia congenita, autosomal dominant													
P12	F	since birth	1	*SCN4A*	NM_000334.4	c.190C > G (p.Leu64Val)	Heterozygote	VUS (PM2, PP3)	22.1	Deleterious (Supporting)	No	Not available	Not available
P13	F	since birth	65	*SCN4A*	NM_000334.4	c.4298 T > G (p.Leu1433Arg)	Heterozygote	Pathogenic (PS4, PP3, PM2, PM1, PP5)	25.2	Deleterious (Strong)	No	Not done	Not done
P14	F	since birth	2	*SCN4A*	NM_000334.4	c.4324G > A (p.Val1442Met)	Heterozygote	Likely pathogenic (PM2, PM1, PP3, PM5)	28.6	Deleterious (Moderate)	No	Not available	Not available
Muscular dystrophy, limb-girdle type 2A (recessive)													
P15	F	early childhood	44	*CAPN3*	NM_000070.2	c.550del (p.Thr184ArgfsTer36)	Homozygote	Pathogenic (PVS1, PM3, PM2, PS3, PP5)	23.8	N/A	No	Not done	the section contained mainly tissue fat overgrown by fibrous connective tissue, in which very few preserved striated muscle fibers were found small diameter and enlarged centrally located nucleus
P16	F	early childhood	66	*CAPN3*	NM_000070.2	c.550del (p.Thr184ArgfsTer36)	Homozygote	Pathogenic (PVS1, PM3, PM2, PS3, PP5)	23.8	N/A	No	100,3 IU/L	primarily muscular changes (biceps)
P17^1^	M	early childhood	16	*CAPN3*	NM_000070.2	c.550del (p.Thr184ArgfsTer36)	Homozygote	Pathogenic (PVS1, PM3, PM2, PS3, PP5)	23.8	N/A	No	5320 U/L	primarily muscle changes of mild intensity (quadriceps femoris muscle)
P18	M	14	37	*CAPN3*	NM_000070.2	c.598_612del (p.Phe200_Leu204del)	Heterozygote compound	Pathogenic (PM3, PM2, PM4, PM1, PP5)	23.4	N/A	No	Not available	Not available
						c.985G > A (p.Gly329Arg)		Pathogenic (PS1, PM3, PM2, PP3, PM1, PP2, PS3, PP5)	29.5	Deleterious (Moderate)	No		
P19^2^	M	Not available	57	*CAPN3*	NM_000070.2	c.(309 + 1_310–1)_(1115 + 1_1116-1)del (exon 2–8 del)	Heterozygote	Pathogenic	N/A	N/A	No	Not available	Not available
						c.319G > A (p.Glu107Lys)	Homozygote	Benign (PP2, BS1, BS2, BP6, BA1)	19.92	Uncertain	No		
Muscular dystrophy, limb-girdle type 2D (recessive)													
P20	F	Not available	5	*SGCA*	NM_000023.3	c.190G > A (p.Ala64Thr)	Heterozygote compound	Likely pathogenic (PM2, PM1, PP2, PP5)	25.2	Uncertain	No	~ 30000 U/L	Not available
						c.574C > T (p.Arg192Ter)		Pathogenic (PVS1, PM3, PM2, PP5)	43	N/A	No		
						c.662G > A (p.Arg221His)		Benign (PP3, PM5, PP2, BP6, BS1, BS2)	28.3	Deleterious (Moderate)	No		
P21	M	3 months	8	*SGCA*	NM_000023.3	c.747G > A (p.Leu249 =)	Heterozygote compound	VUS (PP3, PM2)	21.5	N/A	Yes	23582 U/L 10750 U/L	Not done
						c.790_791dup (p.Gly265GlnfsTer57)		Pathogenic (PVS1, PM3, PM2, PP5)	26.3	N/A	Yes		
P22	M	14	38	*SGCA*	NM_000023.3	c.748G > T (p.Val250Leu)	Heterozygote compound	VUS (PM2, PP3, PP2)	24.3	Uncertain	No	12575 U/L	atrophic fibers of various diameters were found, internalization of the nucleus, excess connective and fatty tissue from degenerative changes, normal dystrophin expression, reduced expression of alpha and gamma sarcoglycans
						c.850C > T (p.Arg284Cys)		Pathogenic (PM3, PM2, PM5, PP3, PP2, PS3, PP1, PP5)	27.4	Deleterious (Supporting)	No		
Muscular dystrophy, limb-girdle type 2B (recessive)													
P23^3^	F	Not available	46	*DYSF*	NM_001130987.2	c.1276 + 5G > A	Heterozygote compound	Pathogenic (PM3, PP3, PM2, PP5)	N/A	N/A	No	6856,3 U/L	features of primarily muscular damage without inflammatory features, changes typical of muscular dystrophy (left quadriceps muscle)
						c.5356del (p.Glu1786ArgfsTer77)		Likely pathogenic (PVS1, PM2)	33	N/A	Yes		
Muscular dystrophy, limb-girdle type 1B (autosomal dominant													
P24	F	Not available	50	*LMNA*	NM_170707.3	c.162_163del (p.Asn56ArgfsTer11)	Heterozygote	Pathogenic (PVS1, PM2, PS4, PP5)	35	N/A	No	Not available	Not available
Muscular dystrophy, Duchenne/Becker muscular dystrophies (X-linked)													
P25	M	Not available	12	*DMD*	NM_004006.2	c.4846-1G > C	Hemizygote	Likely pathogenic (PVS1, PM2, PP5)	33	N/A	Yes	Not available	Not available
P26	M	Not available	9	*DMD*	NM_004006.2	c.6630del (p.Asn2211IlefsTer10)	Hemizygote	Likely pathogenic (PVS1, PM2)	35	N/A	Yes	Not available	Not available
Ullrich congenital muscular dystrophy (AD)													
P27	M	6	51	*COL6A1*	NM_001848.2	c.1029_1032delins	Heterozygote	Likely pathogenic	N/A	N/A	Yes	Not done	fibers of different diameters intermingle, irregularly arranged in bunches. In single fibers nucleus centralization. In a single bunch of lesions under the form overgrowth of connective tissue between fibers, atrophied in this bunch the fibers have rounded shapes
Myopathies													
P28^4^	M	30	45	*RYR1*	NM_000540.2	c.131G > A (p.Arg44His)	Heterozygote compound	Likely pathogenic (PP3, PM2, PM5, PM1, PP2)	28.5	Deleterious (Strong)	No	2797 U/L 4194 U/L	no diagnostic material was obtained for evaluation (right quadriceps muscle)
						c.6523_6525del (p.Glu2175del)		VUS (PM2, PM4, PM1)	20.4	N/A	No		
P29	F	62	73	*RYR1*	NM_000540.2	c.8027G > A (p.Arg2676Gln)	Heterozygote compound	Likely pathogenic (PM2, PM5, PP2, PP5)	23.6	Uncertain	No	769 U/L	features resembling the central core (left quadriceps muscle)
						c.14920C > A (p.His4974Asn)		Likely pathogenic (PM2, PM1, PP3, PP2)	24.1	Deleterious (Supporting)	No		
Nemaline myopathy 6 (AD)													
P30	M	Not available	42	*KBTBD13*	NM_001101362.2	c.1304C > T (p.Ser435Phe)	Heterozygote	VUS (PM2)	25.5	Uncertain	No	Not available	Not available

^1^ – patient 17 had additionally molecularly confirmed DM2;

^2^ – a large deletion was detected by using of MLPA assay in patient 19;

^3^ – patient 23 had additionally molecularly confirmed DM2;

^4^ – patient 28 without molecular testing of DM1 due to the lack of DNA sample

^*^CADD PHRED score calculated for GRCh37-v1.7 using the website https://cadd.gs.washington.edu/score [Schubach M, Maass T, Nazaretyan L, Roner S, Kircher M. *CADD v1.7: Using protein language models, regulatory CNNs and other nucleotide-level scores to improve genome-wide variant predictions.* Nucleic Acids Res. 2023 Nov. 10.1093/nar/gkad989. PubMed PMID: 38183205]. The cut-off was set at 15, where above this value the variant was defined as pathogenic/functional/deleterious and below this value the variant was defined as benign/non-functional/neutral

Myotonia congenita was diagnosed in 14 individuals. Seven different variants were detected in the *CLCN1* gene in either homozygous or compound heterozygous state in 11 patients. Myotonia congenita caused by *SCN4A* mutations was diagnosed in 3 individuals.

The *CLCN1*:c.2680C > T (p.Arg894*) gene is the most frequent pathogenic variant occurs in a homozygous or heterozygous compound state causing autosomal recessive myotonia congenita (Becker disease) in Polish patients. It has been reported in 10 out of 52 studied individuals with an estimated frequency of 19.2%. In five patients it occurred in a homozygous state, whereas in the remaining five cases in a heterozygous compound state with other second variant within this gene.

Variants in 6 different genes were found as a cause of muscular dystrophies. Variants in the *CAPN3* gene resulting in LGMDR1 were identified in 5 patients with an estimated frequency of 9.6%. Seven different variants, including a novel one, were identified in the *SGCA* gene resulting in the diagnosis of LGMDR3. DMD was diagnosed in 2 patients, whereas variants in the *DYSF, LMNA* and *COL6A1* have been found in single patients (Fig. 1B).

The *CAPN3:*c.550del (p.Thr184Argfs*36) gene is the most frequent pathogenic variant causing autosomal recessive limb-girdle muscular dystrophy type 1 (LGMDR1) in Polish patients. It has been reported in 5 out of 52 studied individuals. In three patients it occurred in a homozygous state, which allowed confirmation of LGMDR1. However, in 2 cases it occurred as a single heterozygous variant, which suggest the status of an LGMDR1 carrier.

The *CAPN3*:c.1746-20C > G is widely distributed and previously was known as conflicting interpretation. In this study, its intronic variant was presented both in NMD patients (n = 4) and in the control group (n = 5), reaching an estimated frequency of 7.7% and 2.9%, respectively. Among the 4 affected NMD patients, 2 of them have its intronic variant together with missense variant in the *CAPN3* gene: c.598 T > A (p.Phe200Ile) and c.700G > A (p.Gly234Arg), respectively, whereas the remaining two have a single non-coding *CAPN3* variant. Patient with variants in the *CAPN3*:c.[700G > A];[1746-20C > G] clinically presented calpainopathy: proximal weakness and atrophy, muscle pain, walking difficulties and spine deformities. The calf and quadriceps of the thighs showed muscle hypertrophy. Other symptoms as preserved reflexes, arterial hypertension and dilated cardiomyopathy were observed as well. The EMG examination showed myopathic damage in proximal muscles of the upper and lower limbs without cellular infiltration. The muscle biopsy revealed the presence of dystrophin 10-kDa and 60-kDa, primary muscle damage of a mild neurogenic process, fibers varied in size arranged in bunches and separated discreetly with an increased amount of the connective tissue, internally located nuclei, several splitting fibers and “core-targetoid”, and atrophy fibers. The MRI imaging revealed the atrophy of shoulder and hip girdle muscles. Furthermore, we hypothesize that cardiomyopathy observed in the patient may be partially explained by the presence of the additional heterozygous likely pathogenic variant: c.2243G > C (p.Trp748Ser) in the *POLG* gene, while cardiac abnormalities are rather uncommon for LGMDR1.

In 2 individuals myopathy was associated with variants in the *RYR1* gene, whereas in one individual nemaline myopathy caused by the *KBTBD13* gene was diagnosed.

Among 30 patients presented in Table 1 with a confirmed genetic diagnosis, 27 have already been known, and 4 novel variants have been detected by NGS analysis. The mean age at genetically defined NMD diagnosis was 30 years, whereas the youngest patient was 1 year old, and the oldest one was 73. The shortest delay in diagnosis is several months (Patient 12), while the longest is 65 years (Patient 13).

NGS testing did not identify a definite genetic cause in the remaining 22 patients. In 15 of them, VUS or single pathogenic/likely pathogenic variants were found and summarized in Supplementary Table 2.

### MLPA analysis

The application of MLPA technique towards *CAPN3* gene revealed a heterozygous large deletion of exons 2 – 8 in the *CAPN3* gene in one (Patient 19 with a missense variant c.319G > A (p.Glu107Lys) in the *CAPN3* gene) out of 7 carriers of the single variant within this gene. Patient with a deletion of exons 2 – 8 and c.319G > A (p.Glu107Lys) in the *CAPN3* gene clinically presented proximal muscle weakness of limbs, mild weakness of the girdle, muscle pain, severe muscle cramps, calf hypertrophy, and pes cavus. His laboratory tests revealed an increased level of CK and myoglobin in serum. His EMG study showed primary muscle damage. His two children, brother and parents are unaffected by symptoms of NMDs, whereas the proband and both his brothers and mother are affected by ischemic heart disease.

### Co-existence of two NMDs disorders in single patients

The most common confirmed diseases were channelopathies, followed by muscular dystrophies, and myopathies, which explained together more than half of all our undiagnosed previously NMD cases (Table 1).

Three patients received a genetic diagnosis of DM2. Moreover, in two of them, limb-girdle muscular dystrophy was identified as well, suggesting the possibly more complex inheritance and expression of a phenotype. Patient 17 carried a homozygous frameshift variant known to be pathogenic variant in the *CAPN3* gene together with a heterozygous expansion of CCTG in the *CNBP* gene, corresponding to LGMDR1 calpain3-related disease and DM2. Clinically, he presented muscle weakness and atrophy of the upper and lower limbs, hyperlordosis, scoliosis, foot drop, and a positive Gowers sign. Patient 23 harbored one heterozygous pathogenic and one heterozygous likely pathogenic variants in the *DYSF* gene associated with LGMDR2 dysferlin-related disease as well as a heterozygous expansion in DM2 causing gene.

## Discussion

The complexity of genetic defects associated with NMD and high relative frequency of DM1 and DM2 in Poland, 394 and 441 families respectively [24, current data from studies conducted but not published] necessitated to design a diagnostic approach based on a comprehensive analysis using different molecular techniques. In this study: (1) NGS analysis; (2) DM1 and DM2 genotyping; (3) MLPA assays; and (4) Sanger sequencing have been performed. A targeted 89 NGS gene panel was applied among 52 Polish patients suffering from NMDs. In the tested group, the preliminary clinical diagnoses of myotonia syndromes, muscular dystrophies, or myopathies were established. In total, 29 of them reached a genetic diagnosis after using TGP, placing its effectiveness at 55.8%. Regardless of the NGS data, we identified a dynamic mutation in the *CNBP* gene in three patients and confirmed a gross deletion in the *CAPN3* gene in one individual. Altogether, the diagnostic rate of the established approach reached 57.7% (30 patients).

The most common entity identified in patients was myotonia congenita with variants in the *SCN4A* and *CLCN1* genes. In this study only a recessive form of myotonia congenita caused by pathogenic variants in the *CLCN1* gene was detected. The recent study, evaluating the functional significance of 95 different *CLCN1* variants, suggests that variants resulting in dominant functional features are clustered in the first half of the transmembrane domain and alter voltage dependence of channel activation, whereas variants with recessive functional features without a shift in voltage dependence of activation are clustered in the second half of transmembrane domain of the skeletal muscle chloride channel 1 – CLCN1 protein [24]. Although the c.2680C > T variant has been widely implicated in both dominant and recessive forms of Thomsen-Becker myotonia, according to our results and population frequency data (0.3% in the European non-Finnish population) we suppose that the most common variant c.2680C > T (p.Arg894*) in the *CLCN1* gene cannot be inherited as a dominant one.

One of the common variants of the *CAPN3* gene: c.1746-20C > G was identified as a heterozygous in 4 patients with LGMD phenotype. Its high frequency in Poland has been previously described [25]. Until recently, its intronic variant has been considered a variant with conflicting interpretation of pathogenicity. However, Mroczek et al. (2022) showed that this variant is hypomorphic causing LGMDR1 when occurs in trans position with another pathogenic/likely pathogenic variant [26]. Many studies confirm that this variant is causal when occurs in the compound heterozygous state [25, 27, 28]. According to these findings, we can hypothesize that one of our patients, in whom compound heterozygous *CAPN3:*c.[700G > A];[1746-20C > G] variants together with heterozygous *POLG* likely pathogenic variant: c.2243G > C (p.Trp748Ser) were identified can be diagnosed with LGMDR1. However, to confirm its pathogenicity a segregation analysis in the family is necessary.

In the presented study, a gross deletion encompassing exons 2–8 of the *CAPN3* gene has been also identified by MLPA in a patient, in whom the *CAPN3:*c.319G > A (p.Glu107Lys) variant was found by NGS. *CAPN3:*c.319G > A (p.Glu107Lys) variant has been described previously as a causative pathogenic variant in a heterozygous, compound heterozygous as well as together with variants in the *FKRP* gene [29, 30]. On the other hand, its frequency in the gnomAD database is high and reaches 1.3% within non-Finnish population. Also, numerous ClinVar submitters reported this variant as a benign or likely benign. Based on the literature, databases and our findings we assume that *CAPN3:*c.319G > A identified alone, even in a homozygous state, cannot be classified as a pathogenic one. However, together with another pathogenic variant, it might be implicated in LGMD. To confirm this assumption, a functional study should be performed. Since only a DNA sample was collected from one individual in the family, we have not been able to perform segregation study or functional testing to date. We are aware of this limitation. Here, we aim to note that both variants of the *CAPN3* gene: c.319G > A and deletion of exons 2–8 may together be responsible for the patient’s clinical signs. However, further investigation should be carried out when possible. Moreover, skeletal muscle MRI findings are widely recognized as a useful tool in the diagnosis and clinical management of LGMDR1. Unfortunately, no patient underwent muscle MRI prior to genetic testing. We would like to emphasize that MLPA analysis is worth performing in every patient with the *CAPN3* variant and a questionable diagnosis of LGMD.

Furthermore, during the study, we identified two individuals with co-occurrence of DM2 and LGMD. In one patient, *CNBP* dynamic mutation and *CAPN3* homozygous variant have been detected (Patient 17), whereas in another individual the *CNBP* expansion was present together with *DYSF* variants (Patient 23). Presently, the patient’s phenotype corresponds with LGMDR1 rather than DM2 (Patient 17). The segregation analysis in his family showed that both parents were carriers of a variant in *CAPN3* gene, whereas an expansion in the *CNBP* gene was maternally inherited (Patient 17). In patient 23, the segregation analysis was not available. A similar phenomenon has been already described in several individuals, who harbored point pathogenic variants in the *CLCN1* [31] or *SCN4A* [32] genes together with expansion in the *CNBP* gene, and therefore, all our patients with or without point pathogenic variants in these genes were tested for DM1 and DM2.

In the studied group of 52 patients, the variants in the *CLCN1,* followed by *CAPN3, SCN4A* and *SGCA* genes were most frequently identified. The genetic spectrum of neuromuscular disorders varies, greatly depending on the population and/or country, the size of a tested cohort and their homogeneity or heterogeneity. In the Dutch, the most common genes related to LGMD spectrum were *CAPN3*, *SGCA*/*B*/*G*/*D*, *ANO5* accounting for nearly 70%, whereas the remaining genes included *FKRP*, *EMD*, *GMPPB*, contraction of D4Z4 repeat, *SMN1*, *FLNC*, *MICU1*, *TRIM32* [28]. In Austria, the most frequent cause of limb-girdle muscular weakness and hereditary myopathy were pathogenic variants in *CAPN3*, *FKRP*, *ANO5*, *DYSF*, *SGCA* [33]. However, in China and Turkey the most common cause of LGMD were variants in the *DYSF* and *CAPN3* genes, followed by pathogenic variants in *SGCA*, *LMNA*, and other genes (*DNAJB6*, *FKRP*, *SGCB*, *SGCD*, *TRIM32*, *POMT1*, *ANO5*) [34], and *SGCA*, *CAPN3*, and *DYSF* [35]. Moreover, the presence of homozygous and compound heterozygous variant in the *SGCA* gene: c.850C > T (p.Arg284Cys) reported by Özyilmaz et al. [35] and this study broadens the genetic spectrum of this gene.

Among 32 different variants identified in this study, four are newly discovered and broaden the mutational spectrum of particular genes, including: (1) *DMD*:c.6630del (p.Asn2211Ilefs*10); (2) *COL6A1:*c.1029_1032delinsTTG; (3) *SGCA*:c.747G > A (p.Leu249 =); and (4) *DYSF:*c.5356del (p.Glu1786Argfs*77).

Since all genetic testing methods have their limitations, there is no single comprehensive one, suitable for all purposes. Even advanced techniques such as WES/WGS in some cases may turn to be unavailable. Furthermore, epidemiological factors may also influence a diagnostic strategy. For instance, in some countries DM2 is as prevalent as DM1 or may have a high incidence as in Finland [36]. In Poland, the incidence of DM2 is even higher than DM1 and patients present several unspecific symptoms, according to authors’ published and unpublished data [37]. In the study, we implemented a developed panel to study a group of patients with clinical diagnosis of the spectrum of neuromuscular disorders. The assessment of the targeted gene panel enriched with other methods resulted in effective diagnostics of genetic disorders in this group of patients, expanding the mutational spectrum of the genes implicated in NMDs and maximizing the diagnostic utility.

## Conclusions

1. The application of designed targeted gene panel, involving 89 NMD-causing genes, together with additional techniques (expansion analysis, MLPA assays) was effective and may be useful, particularly when the availability of WES is limited. However, it seems that systematic reanalysis of NGS data, especially as the VUS are concerned, may influence the diagnostic utility.
2. The complexity of the mutational spectrum within the *CAPN3* gene supports the argument that both MLPA and family segregation analysis should be performed in heterozygous or apparently homozygous variant carriers reported with conflicting pathogenicity.
3. Due to reports of coexistence of muscular dystrophy and (CCTG)_n_ repeat expansion in the *CNBP* gene, we suggest considering DM2 and/or DM1 testing also in patients in whom variants of the muscular dystrophy genes have been identified, as it might be related to the phenotype and progression of the disease.

### Supplementary information

Below is the link to the electronic supplementary material.

Supplementary file1 (DOCX 15 KB)

Supplementary file2 (DOCX 23 KB)

## Data Availability

The targeted gene panel sequencing data of this study is not publicly available.

## References

[1] Ozsarlak O, Schepens E, Parizel PM (2001). Hereditary neuromuscular diseases. Eur J Radiol.

[2] Peterlin B, Gualandi F, Maver A (2020). Genetic testing offer for inherited neuromuscular diseases within the EURO-NMD reference network: A European survey study. PLoS ONE.

[3] Gene Table of Neuromuscular Disorders. https://www.musclegenetable.fr/index.html. Accessed 21 Dec 2023

[4] Agrawal PB, Joshi M, Marinakis NS (2014). Expanding the phenotype associated with the NEFL mutation: neuromuscular disease in a family with overlapping myopathic and neurogenic findings. JAMA Neurol.

[5] Calore EE, Cavaliere MJ, Wakamatsu A (1994). An unusual case of muscular limb-girdle dystrophy and mitochondrial myopathy. Pathologica.

[6] Chen RS, Huang CC, Lee CC (1993). Overlapping syndrome of MERRF and MELAS: molecular and neuroradiological studies. Acta Neurol Scand.

[7] Xia Y, Feng Y, Xu L (2021). Case Report: Whole-Exome Sequencing With MLPA Revealed Variants in Two Genes in a Patient With Combined Manifestations of Spinal Muscular Atrophy and Duchenne Muscular Dystrophy [published correction appears in Front Genet. 2021 Jun 01;12:684042]. Front Genet.

[8] Thuriot F, Gravel E, Buote C (2020). Molecular diagnosis of muscular diseases in outpatient clinics: a Canadian perspective. Neurol Genet.

[9] Park J, Oh HM, Park HJ (2019). Usefulness of comprehensive targeted multigene panel sequencing for neuromuscular disorders in Korean patients. Mol Genet Genomic Med.

[10] Beecroft SJ, Yau KS, Allcock RJN (2020). Targeted gene panel use in 2249 neuromuscular patients: the Australasian referral center experience. Ann Clin Transl Neurol.

[11] Gonzalez-Quereda L, Rodriguez MJ, Diaz-Manera J (2020). Targeted next-generation sequencing in a large cohort of genetically undiagnosed patients with neuromuscular disorders in Spain. Genes (Basel).

[12] Krenn M, Tomschik M, Rath J (2020). Genotype-guided diagnostic reassessment after exome sequencing in neuromuscular disorders: experiences with a two-step approach. Eur J Neurol.

[13] Barbosa-Gouveia S, Vázquez-Mosquera ME, González-Vioque E (2022). Rapid molecular diagnosis of genetically inherited neuromuscular disorders using next-generation sequencing technologies. J Clin Med.

[14] Topf A, Johnson K, Bates A, Phillips L, Chao KR, England EM (2020). Sequential targeted exome sequencing of 1001 patients affected by unexplained limb-girdle weakness. Genet Med.

[15] Tsang MHY, Chiu ATG, Kwong BMH (2020). Diagnostic value of whole-exome sequencing in Chinese pediatric-onset neuromuscular patients. Mol Genet Genomic Med.

[16] Waldrop MA, Pastore M, Schrader R (2019). Diagnostic utility of whole exome sequencing in the neuromuscular clinic. Neuropediatrics.

[17] Tian X, Liang WC, Feng Y (2015). Expanding genotype/phenotype of neuromuscular diseases by comprehensive target capture/NGS. Neurol Genet.

[18] Kitamura Y, Kondo E, Urano M (2016). Target resequencing of neuromuscular disease-related genes using next-generation sequencing for patients with undiagnosed early-onset neuromuscular disorders. J Hum Genet.

[19] Neuromuscular Disease Center, Washington University. https://neuromuscular.wustl.edu/syaltbrain.html#muscular. Accessed 17 Dec 2023

[20] Bean LJH, Funke B, Carlston CM, Laboratory Quality Assurance Committee ACMG (2020). Diagnostic gene sequencing panels: from design to report-a technical standard of the American College of Medical Genetics and Genomics (ACMG). Genet Med.

[21] Richards S, Aziz N, Bale S (2015). Standards and guidelines for the interpretation of sequence variants: a joint consensus recommendation of the American College of Medical Genetics and Genomics and the Association for Molecular Pathology. Genet Med.

[22] Brook JD, McCurrach ME, Harley HG (1992). Molecular basis of myotonic dystrophy: expansion of a trinucleotide (CTG) repeat at the 3' end of a transcript encoding a protein kinase family member [published correction appears in Cell. 1992 Apr 17;69(2):385]. Cell.

[23] Warner JP, Barron LH, Goudie D (1996). A general method for the detection of large CAG repeat expansions by fluorescent PCR. J Med Genet.

[24] Suetterlin K, Matthews E, Sud R (2022). Translating genetic and functional data into clinical practice: a series of 223 families with myotonia. Brain.

[25] Macias A, Fichna JP, Topolewska M (2021). Targeted next-generation sequencing reveals mutations in non-coding regions and potential regulatory sequences of Calpain-3 gene in polish limb-girdle muscular dystrophy patients. Front Neurosci.

[26] Mroczek M, Inashkina I, Stavusis J (2022). CAPN3 c.1746-20C>G variant is hypomorphic for LGMD R1 calpain 3-related. Hum Mutat.

[27] Siavrienė E, Petraitytė G, Burnytė B (2021). Compound heterozygous c.598_612del and c.1746–20C > G CAPN3 genotype cause autosomal recessive limb-girdle muscular dystrophy-1: a case report. BMC Musculoskelet Disord.

[28] Ten Dam L, de Visser M, Ginjaar IB (2021). Elucidation of the genetic cause in dutch limb girdle muscular dystrophy families: a 27-year's journey. J Neuromuscul Dis.

[29] Piluso G, Politano L, Aurino S (2005). Extensive scanning of the calpain-3 gene broadens the spectrum of LGMD2A phenotypes. J Med Genet.

[30] Seong MW, Cho A, Park HW (2016). Clinical applications of next-generation sequencing-based gene panel in patients with muscular dystrophy: Korean experience. Clin Genet.

[31] Sun C, Tranebjaerg L, Torbergsen T (2001). Spectrum of CLCN1 mutations in patients with myotonia congenita in Northern Scandinavia [published correction appears in Eur J Hum Genet. 2010 Feb; 18(2):264]. Eur J Hum Genet.

[32] Bugiardini E, Rivolta I, Binda A (2015). SCN4A mutation as modifying factor of myotonic dystrophy type 2 phenotype. Neuromuscul Disord.

[33] Krenn M, Tomschik M, Wagner M (2022). Clinico-genetic spectrum of limb-girdle muscular weakness in Austria: a multicentre cohort study. Eur J Neurol.

[34] Yu M, Zheng Y, Jin S (2017). Mutational spectrum of Chinese LGMD patients by targeted next-generation sequencing. PLoS ONE.

[35] Özyilmaz B, Kirbiyik Ö, Özdemir TR (2019). Impact of next-generation sequencing panels in the evaluation of limb-girdle muscular dystrophies. Ann Hum Genet.

[36] Suominen T, Bachinski LL, Auvinen S (2011). Population frequency of myotonic dystrophy: higher than expected frequency of myotonic dystrophy type 2 (DM2) mutation in Finland. Eur J Hum Genet.

[37] Sulek A, Lusakowska A, Krysa W (2018). Evidence for a relatively high proportion of DM2 mutations in a large group of Polish patients. Neurol Neurochir Pol.

