# Supplementary material for: Next generation sequencing panel as an effective approach to genetic testing in patients with a highly variable phenotype of neuromuscular disorders
Source: Neurogenetics. 2024 May 17;25(3):233–47. doi: 10.1007/s10048-024-00762-y (PMC11249508; doi:10.1007/s10048-024-00762-y)
Supplement: Supplementary file 1 — Supplementary file1 (DOCX 15 KB) [file 10048_2024_762_MOESM1_ESM.docx]

**Supplementary Table 1.** The list of 89 genes associated with NMDs phenotype included in a targeted NGS panel.

| *ACTA1* | *DNM2* | *MYH7* | *TTN* |
| --- | --- | --- | --- |
| *AGRN* | *DOK7* | *MYOT* | *VCP* |
| *ANO5* | *DPAGT1* | *NEB* |  |
| *ATP2A1* | *DPM2* | *PLEC* |  |
| *B3GNT1* | *DYSF* | *POMGNT1* |  |
| *BAG3* | *EMD* | *POMGNT2* |  |
| *BIN1* | *FHL1* | *POMT1* |  |
| *CACNA1S* | *FKRP* | *POMT2* |  |
| *CAPN3* | *FKTN* | *PTRF* |  |
| *CAV3* | *FLNC* | *RAPSN* |  |
| *CCDC78* | *GFPT1* | *RYR1* |  |
| *CFL2* | *GNE* | *SCN4A* |  |
| *CHAT* | *HSPG2* | *SEPN1* |  |
| *CHKB* | *HINT1* | *SGCA* |  |
| *CHRNA1* | *ISPD* | *SGCB* |  |
| *CHRNB1* | *ITGA7* | *SGCD* |  |
| *CHRND* | *KBTBD13* | *SGCG* |  |
| *CHRNE* | *KCNJ2* | *SMCHD1* |  |
| *CLCN1* | *KLHL40* | *SYNE1* |  |
| *CNTN1* | *LAMA2* | *SYNE2* |  |
| *COL6A1* | *LARGE* | *TCAP* |  |
| *COL6A2* | *LDB3* | *TIA1* |  |
| *COL6A3* | *LMNA* | *TMEM5* |  |
| *COLQ* | *MATR3* | *TMEM43* |  |
| *CRYAB* | *MEGF10* | *TNNT1* |  |
| *DAG1* | *MTM1* | *TPM2* |  |
| *DES* | *MUSK* | *TPM3* |  |
| *DMD* | *MYBPC3* | *TRAPPC11* |  |
| *DNAJB6* | *MYH2* | *TRIM32* |  |
