# Supplementary material for: Next generation sequencing panel as an effective approach to genetic testing in patients with a highly variable phenotype of neuromuscular disorders
Source: Neurogenetics. 2024 May 17;25(3):233–47. doi: 10.1007/s10048-024-00762-y (PMC11249508; doi:10.1007/s10048-024-00762-y)
Supplement: Supplementary file 2 — Supplementary file2 (DOCX 23 KB) [file 10048_2024_762_MOESM2_ESM.docx]

**Supplementary Table 2**. Summary of 15 patients with neuromuscular disorders, in whom VUS or likely pathogenic/pathogenic variants were identified after using a targeted NGS gene panel. The phenotype of the patients was not consistent with genetic findings or only a single variant or VUS was detected.

| **Patient ID** | **Sex** | **Gene** | **Variants** | **ACMG score** | **Phenotype (Inheritance)** | **Novel** | **References** | **Comments** |
| --- | --- | --- | --- | --- | --- | --- | --- | --- |
| P31 | F | *CAPN3* | NM_000070.3:c.550delA  NP_000061.1:p.(Thr184ArgfsTer36) | Pathogenic | Muscular dystrophy, limb-girdle type 1I (AD) | No | Richard et al. 1995 | Some variant carriers may be asymptomatic. The variant c.550delA is the most common and reported in Polish families caused by homozygous or compound heterozygous variant. |
| P32 | F | *CAPN3* | NM_000070.3:c.550delA  NP_000061.1:p.(Thr184ArgfsTer36) | Pathogenic |  |  |  |  |
| P33 | M | *CAPN3* | NM_000070.3:c.700G>A  NP_000061.1:p.(Gly234Arg) | VUS |  | No | Dorobek et al. 2015 | Some variant carriers may be asymptomatic. |
| P34 | F | *POMT2* | NM_013382.7:c.551C>T  NP_037514.2:p.(Thr184Met) | Likely pathogenic | Muscular dystrophy-dystroglycanopathy OMIM:607439 (AR) | No | Godfrey et al. 2007 | Genotype-phenotype matching. |
| P35 | F | *TTN* | NM_001267550.2:c.102073G>C  NP_001254479.2:p.(Glu34025Gln) | VUS | Muscular dystrophy, limb-girdle, type 2J (AR) |  |  | A 34-year-old patient suspected with muscular dystrophy and negative family history. It seems that phenotype is consistent with genotype. |
|  |  |  | NM_001267550.2:c.52751C>T NP_001254479.2:p.(Thr17584Ile) | VUS |  |  |  |  |
|  |  |  | NM_001267550.2:c.75526C>T  NP_001254479.2:p.(Arg25176Cys) | VUS |  |  |  |  |
| P36 | F | *DOK7* | NM_173660.5:c.1511_1513del NP_775931.3:p.(Pro504delins) | Likely pathogenic | Myasthenic syndrome, congenital (AR) |  |  | Phenotype do not match to genotype. A 25-year old patient suspected with muscular dystrophy and negative family history. |
|  |  |  | NM_001301071.2:c.1526C>T NP_001288000.1:p.(Ala509Val) | Likely pathogenic |  |  |  |  |
| P37 | F | *ATP2A1* | NM_173201.5:c.841G>A  NP_775293.1:p.(Asp281Asn) | Likely pathogenic | Brody myopathy (AR) |  |  | A 33-year old patient with negative family history. Only a single heterozygous Variant in a recessive disease was detected. |
| P38 | M | *PLEC* | NM_201380.4:c.1778T>C  NP_958782.1:p.(Val593Ala) | Likely pathogenic | Muscular dystrophy, limb-girdle, type 2Q (AR) |  |  | It seems that phenotype is consistent with genotype, however only a single heterozygous variant was found. |
| P39 | M | *TTN* | NM_001267550.2:c.89725A>G  NP_001254479.2:p.(Lys29909Glu) | VUS | Muscular dystrophy, limb-girdle, type 2J (AR) |  |  | It seems that phenotype is consistent with genotype. |
| P40 | M | *FKRP* | NM_024301.5:c.1379A>G  NP_077277.1:p.(Gln460Arg) | VUS | Muscular dystrophy, limb-girdle, type 2I (AR) |  |  | Phenotype do not match to genotype. A 49-year old patient suspected with myotonia and negative family history. |
|  |  | *TTN* | NM_001267550.2:c.40681A>G  NP_001254479.2:p.(Thr13561Ala) | VUS | Muscular dystrophy, limb-girdle, type 2J (AR) |  |  |  |
|  |  | *AGRN* | NM_198576.4:c.2241G>C  NP_940978.2:p.(Gln747His) | VUS | Myasthenic syndrome, congenital (AR) |  |  |  |
| P41 | F | *NEB* | NM_001271208.2:c.22236C>G  NP_001258137.2:p.(His7412Gln) | VUS | Nemaline myopathy 2 (AR) |  |  | A 40 year-old patient with negative family history. |
| P42 | M | *CHAT* | NM_020549.5:c.70G>A NP_065574.4:p.(Gly24Ser) | VUS | Myasthenic syndrome, congenital (AR) |  |  | A 48 year-old patient suspected with myopathy. Additionally, a single heterozygous variant c.1399G>A (p.Ala467Thr) in *POLG* gene was detected. |
|  |  | *COL6A3* | NM_004369.4:c.4828A>G  NP_004360.2:p.(Met1610Val) | VUS | Bethlem myopathy 1 (AD) |  |  |  |
|  |  | *DMD* | NM_004006.3:c.2391T>G  NP_003997.2:p.(Asn797Lys) | VUS | Muscular dystrophy OMIM:300377 (AR) |  |  | In LOVD database classified as pathogenic, VUS, likely benign and benign. |
| P43^1^ | M | *DYSF* | NM_001130987.2:c.1271C>T  NP_001124459.1:p.(Pro424Leu) | VUS | Muscular dystrophy / myopathy OMIM:603009 (AR) |  |  | Phenotype do not match to genotype. A 49 year old patient suspected with myotonia Thomsena. |
|  |  | *TTN* | NM_001267550.2:c.49937G>A  NP_001254479.2:p.(Arg16646Gln) | VUS | Muscular dystrophy, limb-girdle, type 2J (AR) |  |  |  |
|  |  |  | NM_001267550.2:c.3100G>A  NP_001254479.2:p.(Val1034Met) | VUS |  |  |  | In LOVD database classified as likely pathogenic, VUS, likely benign. |
| P44 | F | *RYR1* | NM_000540.3:c.14920C>A  NP_000531.2:p.(His4974Asn) | Likely pathogenic | Myopathy OMIM:180901 (AD, AR) |  |  |  |
|  |  |  | NM_000540.3:c.8027G>A NP_000531.2:p.(Arg2676Gln) | VUS |  |  |  |  |
|  |  | *SELENON* | NM_020451.3:c.1397G>A | Likely pathogenic | Muscular dystrophy (AR) / myopathy (AD, AR) |  |  |  |
| P45 | M | *MYH7* | NM_000257.4:c.2348G>A  NP_000248.2:p.(Arg783His) | VUS | Myopathy (AD, AR) |  |  | Phenotype do not match genotype. A 7-year-old patient suspected with muscular dystrophy. |

^1^ patient P43, with genetic confirmation of DM2
